# A survey study of medical knowledge about anaphylaxis

**DOI:** 10.1186/1939-4551-8-S1-A229

**Published:** 2015-04-08

**Authors:** Marinauria Leal Pinto, Mara Morelo Rocha Felix, Cintia Bordalo, Sônia Hoana Silva, Karla Do Carmo Ferrão, Monica De Britto Pereira Bandeira De Mello, Jaqueline Ribeiro Toscano De Brito, Raquel Grinapel, Aniela Bonorino Xexeo Castelo Branco, Jaqueline Coser Vianna, Monica Soares De Souza, Andreia Albuquerque Garcês

**Affiliations:** 1 Federal Hospital of Servidores Do Estado, Brazil

## Background

Anaphylaxis is a life-threatening, systemic hypersensitivity reaction, usually IgE mediated. It is the most severe form of allergic reaction and is almost always unexpected. Delay in treatment results in death by airway obstruction or vascular collapse.

## Methods

We conducted an observational study with questionnaires about etiology, diagnosis and treatment of anaphylaxis between April and July 2014 at the Federal Hospital of Servidores do Estado/RJ.

## Results

We evaluated 80 questionnaires answered by physicians (38 male) from various specialties. The majority of them were between 20 and 30 years-old. The most frequent specialties were Orthopedics and Pediatrics (22.5%). The graduate year was between 2005 and 2013 in 62.5%. Forty-six had already treated patients with anaphylaxis. The most frequent triggers were: drugs (22), food (20), insect stings (2) and others (2). The drugs cited were: NSAIDs (21), beta-lactam antibiotics (13), contrast media (9), NMBAs (2), latex (1), local anesthesics (1), colloids (1), captopril (1), and SMX-TMP (1). Food implicated were: sea food (16), shrimp (11), fish (4), milk (4), peanut (3) and egg (2). About first choice treatment of anaphylaxis, 36.3% answered that it was epinephrine IM, 32.5% said epinephrine SC, 13.8% corticosteroid IV, 10% epinephrine IV, and 2.5% antihistamines. Eighty-five percent of respondents referred those patients with anaphylaxis to allergists. Pediatricians treated the majority of cases of anaphylaxis (9), most of them caused by food. Pediatricians and physicians with more recent year of graduation cited the epinephrine IM as the appropriate treatment more often.

## Conclusions

Anaphylaxis occurs as the result of allergen response, usually IgE mediated, which leads to mast cells and basophils activation and a combination of cutaneous, respiratory, cardiovascular, gastrointestinal, and neurological symptoms. Cutaneous and respiratory symptoms are the most common. The three most common triggers are food, insect stings and drugs. Epidemiological research is necessary for estimating the true prevalence and mortality. Education of health professionals is indispensable for recognition and management of anaphylaxis.

